# Delayed endodontic management of traumatically exposed immature maxillary anterior teeth: a case series

**DOI:** 10.2340/biid.v13.45434

**Published:** 2026-02-26

**Authors:** Vignesh Ravi, Ruchi Singhal, Madhur Sharma, Ritu Namdev

**Affiliations:** a Department of Pediatric and Preventive Dentistry, Post Graduate Institute of Dental Sciences, Pandit Bhagwat Dayal Sharma University, of Health Sciences, Rohtak, Haryana, India; b Department of Oral and Maxillofacial Pathology, Post Graduate Institute of Dental Sciences, Pandit Bhagwat Dayal Sharma University of Health Sciences, Rohtak, Haryana, India

**Keywords:** Pulpotomy, apexogenesis, young teeth, complicated crown fracture, trauma

## Abstract

**Background:**

Traumatic dental injuries are a leading cause of pulpal exposure in immature permanent anterior teeth. Vital pulp therapy (VPT), particularly pulpotomy, has become the preferred treatment modality in such cases as it maintains pulp function, supports physiologic dentin deposition, and promotes long-term tooth retention. However, treatment delays may compromise clinical outcomes due to bacterial contamination and progressive pulpal inflammation. Mineral trioxide aggregate (MTA) and Biodentine™, both hydraulic calcium silicate cements, have demonstrated favorable biological responses in VPT.

**Aim:**

To evaluate clinical and radiographic outcomes of full pulpotomy using MTA and Biodentine™ in immature permanent teeth with complicated crown fractures and delayed treatment intervals of up to 10 days.

**Methods:**

This case series included eight immature maxillary central incisors in seven patients, aged 8.5–9.5 years, presenting with complicated crown fractures. Full pulpotomy was performed using either MTA or Biodentine™ following standardized clinical protocols, and teeth were restored in the same visit, with fragment reattachment performed when feasible. Clinical and radiographic follow-up was conducted at 1 week and at 1, 3, 6, 12, 18, and 24 months.

**Results:**

At 18–24 months, all treated teeth remained vital, demonstrated resolution of symptoms, and showed continued physiologic root development consistent with successful apexogenesis. A radiographic dentinal bridge was observed in three teeth. No cases exhibited pathological changes such as periapical radiolucency, swelling, or restoration failure, except for one case with crown discoloration.

**Conclusion:**

Full pulpotomy using MTA or Biodentine™ may represent a promising treatment option for traumatically exposed immature permanent teeth, even when initiated up to 10 days after injury; however, these findings should be interpreted cautiously in view of the limited sample size and follow-up duration.

## Introduction

Dental trauma is frequently encountered in the permanent dentition and may occur at any age [1], with peak incidence reported between the ages of 2–3 and 8–12 years [2]. The condition is more prevalent among males. Falls, road accidents, interpersonal violence, domestic incidents, and sports activities are the common causes of traumatic dental injuries (TDIs) [2]. Crown fractures constitute approximately 8.5% to 34.5% of all TDIs [3]. Treatment options for crown fractures depend on pulpal involvement. Uncomplicated crown fractures are typically restored using direct composite restorations or fragment reattachment, whereas complicated fractures often require vital pulp therapy (VPT), including direct pulp capping or partial/full pulpotomy. In cases of pulpal necrosis, treatment options include revascularization, apexification, or conventional root canal therapy (RCT), depending on the status of the root maturity [1].

Preservation of pulp vitality in young permanent teeth is crucial, as it prevents bacterial invasion [4], supports natural root development, promotes a favorable crown-to-root ratio, and results in thick dentinal walls which are essential for long-term structural integrity and function [5]. Prognosis depends on several factors, including root development stage, extent of injury, condition of the periodontal ligament and apical vasculature, presence of concomitant injuries, timing and adequacy of intervention, type of medicament, and patient compliance with follow-up care [4].

Historically, the size of pulp exposure has guided treatment planning [6]. However, emerging evidence indicates that exposure size alone does not significantly influence pulpotomy outcomes [7]. Timeliness of treatment remains a key prognostic determinant. Delays exceeding 24–72 h increase bacterial contamination, accelerating pulpal deterioration and reducing the likelihood of successful healing and dentinal bridge formation [6, 7]. Also, pulpal necrosis is more likely to occur in teeth with crown fractures accompanied by luxation injuries [3, 7]. Calcium hydroxide has long served as the conventional pulp-capping material, although its reported success rates have been inconsistent [3]. Hence, hydraulic calcium silicate cements (HCSC) such as mineral trioxide aggregate (MTA) and Biodentine™ have gained preference due to their superior biocompatibility, reliable sealing, and favorable biological responses, alongside practical advantages in handling and setting characteristics [4, 5].

Clinical management of traumatized teeth is further influenced by patient-related challenges. Children, who constitute the majority of cases with complicated crown fractures, commonly exhibit dental anxiety and limited tolerance for prolonged procedures [8, 9]. Shorter treatment sessions, therefore, offer a significant advantage in pediatric care, as they can enhance cooperation and improve compliance [10].

Loss of pulpal vitality in immature teeth complicates endodontic management due to incomplete root development and thin dentinal walls, predisposing the tooth to structural failure and negatively affecting long-term prognosis [11]. Therefore, whenever feasible, maintaining pulpal vitality is preferable to non-vital treatment modalities [12].

Thus, the aim of this study was to present and evaluate the clinical and radiographic outcomes of full pulpotomy using MTA and Biodentine™ in traumatically exposed immature permanent teeth with delayed treatment intervals of up to 10 days, in accordance with the International Association of Dental Traumatology (IADT) guidelines 2020 [13].

## Case presentation

A total of eight immature permanent maxillary central incisors from seven patients aged 8.5–9.5 years, all presenting with clinically and radiographically confirmed complicated crown fractures and a trauma history of 5–10 days, were included in this study. All patients presented with moderate to severe post-traumatic sensitivity or pain and were self-medicating with over-the-counter analgesics.

Pulp sensibility testing was performed using a cold test and an electric pulp test (EPT). Teeth were isolated and dried using cotton rolls. For the cold test, a cotton pellet sprayed with refrigerant spray (Endo Ice F, Coltene/Whaledent, USA) was applied to the labial surface. For the EPT, the tooth was isolated and dried, and a conducting medium (toothpaste) was applied to the electrode tip before placement on the labial surface. The current was gradually increased, and patients were instructed to report the first sensation of tingling, warmth, or pain. Responses were compared with contralateral non-traumatized control teeth. All traumatized teeth demonstrated hypersensitive responses. Medical histories were non-contributory. Post-traumatic pain and sensitivity were categorized using Wolters’ classification [14]. Clinical characteristics of the participants are summarized in Table 1.

**Table 1:** Clinical characteristics.

Case	Age / gender	Tooth number*	Root formation stage#	Type of trauma	Duration of pulp exposure	Size of pulp exposure	Associated periodontal injury	Wolter’s classification	Diagnosis	Type of pulpotomy	Bleeding time	Type of medicament	Final restoration	Follow-up duration
Case-1	9.5 / M	11	Stage 3	Mechanical-RTA	7 days	2 mm	None	Severe	CCF	Full	4 mins	Biodentine™	Fragment reattachment	24 months
Case-2	9 / M	11	Stage 3	Mechanical-sports	5 days	1.5 mm	None	Moderate	CCF	Full	6 mins	MTA	Fragment reattachment	18 months
Case-3	9.5 / F	21	Stage 4	Mechanical- sports	5 days	1.5 mm	Subluxation	Severe	CCF+S	Full	4 mins	MTA	Fragment reattachment	18 months
Case-4	9 / M	21	Stage 4	Mechanical- sports	10 days	3 mm	None	Moderate	CCF	Full	3 mins	MTA	Composite	24 months
Case-5	8.5 / M	11	Stage 3	Mechanical- RTA	7 days	1.5 mm	Subluxation	Moderate	CCF+S	Full	5 mins	Biodentine™	Composite	24 months
Case-6	9 / M	11 & 21	Stage 3	Mechanical- sports	8 days	2.5 mm	None	Moderate	CCF	Full	4 mins	MTA	Composite	24 months
Case-7	9 / M	11	Stage 4	Mechanical- sports	10 days	2 mm	None	Severe	CCF	Full	5 mins	Biodentine™	Composite	18 months

M: male; F: female; RTA: road traffic accident; CCF: complicated crown fracture; S: subluxation; MTA: mineral trioxide aggregate.

* ADA numbering system.

# Cvek’s classification of root development.

### Treatment plan

Pulpotomy with MTA or Biodentine™ was planned. The procedure, potential risks, and expected clinical course were explained to patients and guardians. Written informed consent and assent were obtained.

All VPT procedures were performed by a single operator (pedodontist). Local anesthesia was administered using 2% lidocaine with 1:100,000 epinephrine (ICPA Health Products Ltd., Ankleshwar, India), followed by rubber dam isolation. Although a partial pulpotomy was initially planned, due to clinical factors such as persistent bleeding, extensive crown loss, or the presence of superficial pulpal necrosis, a full pulpotomy was ultimately performed in all cases. Full pulpotomy was performed using a high-speed size 3 round diamond bur under copious water coolant, removing the exposed pulp tissue till the canal orifice. Tissue removal proceeded until healthy pulp was evident, characterized by controlled bleeding within a clinically acceptable period and normal color and consistency.

The exposed pulp surface was disinfected with 2.5% sodium hypochlorite. A cotton pellet soaked in the same solution was applied to control bleeding and further disinfect the site, with reassessment at 1-min intervals. MTA (MTA Angelus® White, Angelus, Londrina, Paraná, Brazil) or Biodentine™ (Septodont, Saint-Maur-des-Fossés, France) was mixed as per manufacturer instructions and placed in the pulp chamber. A saline-moistened cotton pellet was positioned for 20 min to support the initial hydration phase, as both materials are HCSCs requiring moisture for optimal setting [15]. Subsequently, the area was sealed with glass ionomer cement (Vitrebond, 3M Dental Products Division, St. Paul, MN, USA), followed by application of adhesive (Adper Single Bond 2; 3M ESPE, St. Paul, MN, USA) and composite resin (Valux Plus; 3M ESPE, St. Paul, MN, USA). When available, fractured tooth fragments were rehydrated in a sodium fluoride solution for a minimum of 60 min and reattached using a flowable composite (Tetric Flow, Ivoclar Vivadent AG, Schaan, Liechtenstein) in the same visit [16, 17].

### Follow-up

**Figure 1:**
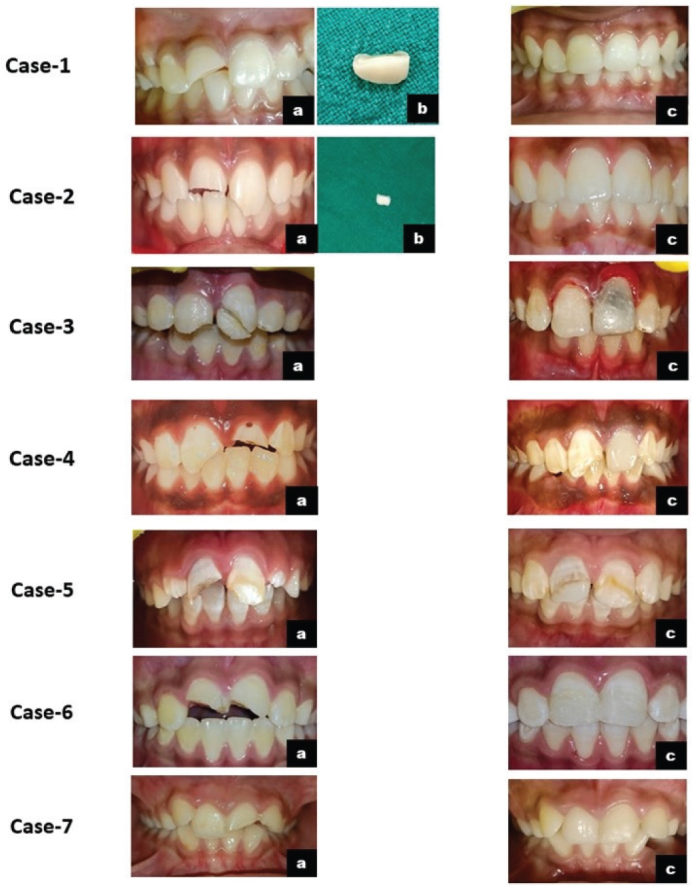
Intra-oral photographs of cases 1–7. a- pre-operative; b- fracture tooth segment; c-post-operative at follow-ups. 3-c showing discoloration of 21.

**Figure 2:**
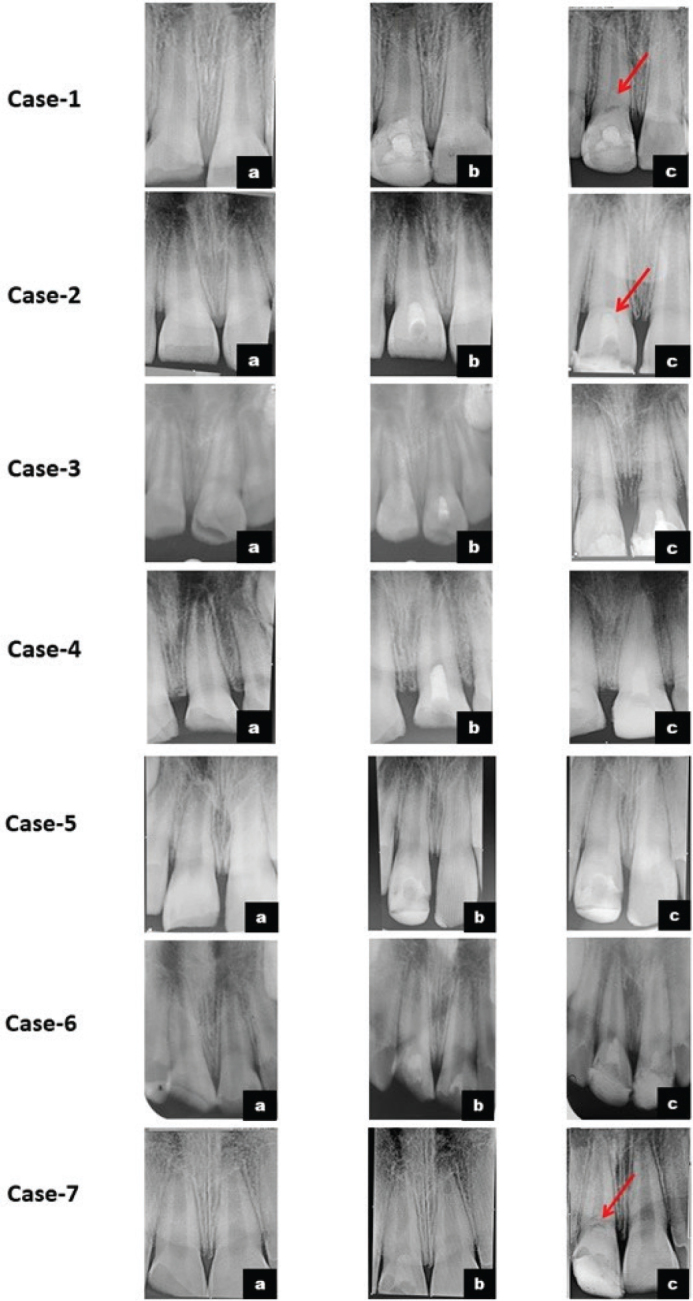
Intra-oral periapical radiographs of cases 1–7. a- pre-operative; b- immediate post-operative; c- post-operative at last follow-ups with evidence of continued apexogenesis; red arrow marks dentin bridge formation.

Patients were recalled at 1 week, and at 1, 3, 6, 12, 18, and 24 months. Cases were classified as failures if any adverse clinical signs or symptoms developed during the follow-up period. Teeth were assessed for abnormal signs and symptoms such as spontaneous pain, hypersensitivity, chewing discomfort, swelling, mobility, and restoration integrity. Pulp vitality tests were performed at each follow-up visit and showed positive responses in all cases. All restorations were esthetically acceptable and functionally sound, except for one tooth that showed discoloration (Figure 1). Radiographic assessment confirmed dentin bridge formation in three cases. At recall, all teeth were asymptomatic, demonstrating favorable radiographic healing and physiological apical development (Figure 2). Post-operative outcomes, dentin bridge formation, and results of vitality testing are summarized in Table 2. This study was based on previously documented clinical cases managed by the Pediatric and Preventive Dentistry, for which informed consent had already been obtained. All patient information was anonymized to ensure complete confidentiality. Therefore, separate institutional ethical approval was not mandated. Given the limited sample size, no statistical testing was performed, and outcomes were documented descriptively. This approach aligns with methodological norms for early-stage clinical case series designed to generate hypotheses rather than produce inferential statistical conclusions.

**Table 2:** Clinical and radiological outcomes of participants.

Case	Follow-up duration	Clinical				Radiological		
		Quality of restoration at follow-up	Clinical symptoms	Vitality	Discoloration	Radiographic pathology	Dentin bridge formation	Root completion
**Case-1**	24 months	✓	–	+	–	–	+	+
**Case-2**	18 months	✓	–	+	–	–	+	+
**Case-3**	18 months	✓	–	+	+	–	–	+
**Case-4**	24 months	✓	–	+	–	–	–	+
**Case-5**	24 months	✓	–	+	–	–	–	+
**Case-6**	24 months	✓	–	+	–	–	–	+
**Case-7**	18 months	✓	–	+	–	–	+	+

‘–’- negative/absent; ‘+’- positive/present; ‘✓’- sound, intact and functional.

## Discussion

A viable Hertwig’s epithelial root sheath (HERS) is essential for continued root development [18]. Any trauma during the formative stage will jeopardize the pulpal blood and nerve supply, leading to pulp necrosis and apical periodontitis; impairing root development, causing a negative impact on tooth survival and the long-term prognosis [18, 19]. Following traumatic injury, primary odontoblasts typically degenerate, after which the pulp initiates reparative dentinogenesis through the recruitment of stem or progenitor cells that differentiate into odontoblast-like cells. The dentin matrix itself serves as a reservoir of bioactive components – such as growth factors, cytokines, and matrix proteins – that can stimulate either surviving odontoblasts or newly differentiated odontoblast-like cells, leading to the deposition of a new dentinal matrix [4, 20].

Current IADT guidelines support VPT for traumatic pulp exposure in young permanent teeth; however, the lack of explicit clinical criteria necessitates individualized treatment decisions based on pulpal inflammatory status, biological response, and predicted healing potential [13]. Immature apices, smaller pulp exposures, and absence of concomitant periodontal trauma are generally associated with favorable outcomes, whereas delayed intervention, complex coronal fracture patterns, failure of restoration, and mature root development correlate with reduced success [21]. Ultimately, successful pulp preservation depends on intraoperative evaluation of pulpal bleeding and tissue quality, rather than preoperative predictors alone.

In the present case series, full pulpotomy was selected based on clinical presentation rather than default treatment philosophy. Uncontrolled hemorrhage during attempted partial pulpotomy [22], inadequate remaining tooth structure [23], and the presence of localized superficial necrosis necessitated the complete removal of the coronal pulp. Additionally, the extended time lapse between trauma and treatment may increase the likelihood of salivary and bacterial contamination of the pulp in complicated crown fractures [7]. Research by Cvek et al. suggests that within 48 h, the inflammatory reaction typically extends no further than two millimetres from the exposed pulp surface; however, prolonged exposure may result in deeper inflammation [24].

Small pulpal exposures (< 1 mm) are usually managed by conservative pulp treatments, and larger exposures are frequently assumed to be associated with increased pulp necrosis risk and inflammation [24]. However, Fuks and colleagues reported that exposure size does not affect treatment outcomes as long as residual pulp tissue remains vital, demonstrating success rates of 94% and 87.5% [25, 26]. Consistent with these findings, the present study also demonstrated that size of pulp exposure did not influence the overall treatment outcome.

Delays between the occurrence of a fracture and the initiation of treatment are common, with studies indicating that only 43% to 54% of patients receive treatment on the day of injury [6]. Studies by Fuks et al. [27] and Gelbier & Winter et al. [28], revealed a strong correlation between pulp necrosis and a lower incidence of hard tissue barrier formation, when therapy was delayed more than 24 h after the injury [29]. However, two other studies reported no significant reduction in pulpotomy success with a delay in intervention, and high success rates were achieved [30, 31]. These findings align with current evidence indicating that treatment delay does not adversely affect the success of VPT, suggesting that exposure size and timing may not be absolute determinants and should instead be interpreted in conjunction with broader clinical parameters [32, 33].

Concomitant traumatic injuries have been shown to exert a detrimental effect on pulpal healing, resulting in poorer outcomes compared with isolated complicated crown fractures [13, 30]. Haikal et al. [3] identified associated luxation trauma as the primary independent factor reducing treatment success, while Shahmohammadi et al. [34] and Tzanetakis GN et al. [35] found that complicated crown fractures accompanied by concussion or subluxation showed a higher risk of pulp necrosis. Notably, in the present study, two patients who presented with concomitant subluxation demonstrated healthy pulpal status and continued root development at the end of the follow-up period. This supports the view that although luxation may heighten the risk of pulp necrosis, it does not contraindicate VPT [6]. However, vigilant follow-up remains essential for the timely detection of pulp necrosis and appropriate management.

The sign of a healthy, functional pulp is the synthesis of physiological dentin, forming a dentinal bridge. HCSCs continuously release Ca^2+^ ions, which reduce capillary permeability, fluid leakage, and intercellular fluid volume while concurrently raising extracellular Ca^2+^ levels, reducing pulpal inflammation, and maintaining the mineralization process. These effects work together to create a favorable milieu that promotes the adhesion, migration, differentiation, and proliferation of stem/progenitor pulp cells. The high alkalinity of HCSCs enhances collagen production by pulp-derived cells and upregulates calcification-related enzymes such as alkaline phosphatase (ALP). Upon contact with pulpal fluid, these materials release calcium hydroxide, which dissociates to liberate calcium ions that subsequently react with phosphate ions to form hydroxyapatite crystals. The porous structure of the cements facilitates continuous hydroxyapatite deposition, and as the material gradually dissolves, hydroxyapatite nucleates and accumulates on its surface, ultimately resulting in the formation of a thick, structurally continuous dentinal bridge at the HCSC–pulp interface [18, 20]. The dentinal bridge produced by calcium hydroxide has porosities and defects that allow bacteria to directly access the pulp, whereas the HCSCs MTA and Biodentine™ create a thicker and higher quality hard tissue barrier [7, 36, 37]. In three of the present cases, the radiographs showed a dentinal bridge as a radiopaque zone beneath MTA and Biodentine™.

Previous studies have shown favorable outcomes for HCSC based pulpotomy materials, with Biodentine achieving a 91% success rate at 15 months and MTA demonstrating an 81.5% healing rate over 36 months while maintaining pulp vitality and continued root development in immature permanent teeth [3, 38]. Abuelniel et al. [5] reported outcomes for MTA and Biodentine®, with 80% clinical success rate and radiographic success rate of 84% and 80% respectively, and no significant difference in overall success rate. Systematic reviews have demonstrated favorable outcomes with these bioactive materials in the management of complicated crown fractures, supporting their role in maintaining pulp vitality and promoting continued root development [7]. The favorable clinical and radiographic outcomes observed in this study may be attributed to the beneficial physical and bioactive properties of MTA and Biodentine™, consistent with findings reported in previous studies.

Evaluating the condition of the injured pulp is crucial to provide a baseline for subsequent follow-up assessments [22]. The pulp’s ability to regain its vitality is contingent upon several factors, as mentioned earlier [2]. In our study, the traumatized tooth responded comparable to the contralateral tooth on cold testing and EPT during the follow-up period. Given the challenges and unreliability of clinically identifying vital pulp tissue, evaluation of periapical health was considered radiographically a relevant outcome [6, 22]. This was corroborative with our study that all the teeth showed completed apexogenesis of open apices. This indicates vital pulp with continued normal physiological activity in the apical region.

All these findings support the biological potential of the pulp to heal under appropriate aseptic and biomimetic conditions, emphasizing that the pulpal and periodontal status, and use of biocompatible materials are key determinants of successful apexogenesis.

### Limitations

The primary limitations of this case series include the small sample size and study design, which may limit the generalizability of the findings. There is a need for a larger prospective cohort or multicenter observational studies with longer follow-up periods to confirm the effectiveness of MTA and Biodentine™ as pulpotomy materials and also to clearly define the influence of treatment delay and type of pulpotomy approach on clinical outcomes in immature teeth.

## Conclusion

In this case series, full pulpotomy with MTA or Biodentine™ was associated with favorable clinical and radiographic outcomes up to 18–24 months in immature maxillary incisors presenting 5–10 days after trauma. These results are preliminary; larger prospective studies with standardized outcome measures and longer follow-up are required to confirm efficacy in delayed presentations.
